# Authority matters: propaganda and the coevolution of behaviour and attitudes

**DOI:** 10.1017/ehs.2022.48

**Published:** 2022-10-28

**Authors:** Sergey Gavrilets, Peter J. Richerson

**Affiliations:** 1 Department of Ecology and Evolutionary Biology, Department of Mathematics, Center for the Dynamics of Social Complexity, University of Tennessee, Knoxville, TN 37996, USA; 2 Department of Environmental Science and Policy, University of California, Davis, CA 95616, USA

**Keywords:** Cooperation, beliefs, inculcation, modelling, identity

## Abstract

Human decision-making is controlled by various factors including material cost–benefit considerations, values and beliefs, social influences, cognitive factors and errors. Among social influences, those by external authorities (e.g. educational, cultural, religious, political, administrative, etc.) are particularly important owing to their potential reach and power. To better understand the effects of ‘soft’ power of authorities we develop a unifying theoretical framework integrating material, cognitive and social forces controlling the joint dynamics of individual actions and beliefs. We apply our approach to three different phenomena: evolution of food sharing in small-scale societies, participation in political protests and effects of priming social identity in behavioural experiments. For each of these applications, we show that our approach leads to different (or simpler) explanations of human behaviour than alternatives. We highlight the type of measurements which can be helpful in developing practical applications of our approach. We identify and explicitly characterise the degree of mismatch between individual actions and attitudes. We assert that the effects of external authorities, of changing beliefs and of differences between people must be studied empirically, included in mathematical models, and accounted for when developing different policies aiming to modify or sustain human behaviour.

## Graphical abstract

**Figure:**
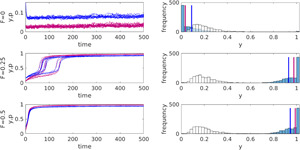

**Social media summary**. A theoretical study of the power of inculcation, propaganda and social identity in shaping human behaviour and beliefs.

… a belief constantly inculcated during the early years of life, whilst the brain is impressible, appears to acquire almost the nature of an instinct; and the very essence of an instinct is that it is followed independently of reason. (Darwin, 1871: 82)

What did you dream?

It's alright, we told you what to dream. (Pink Floyd, ‘Welcome to the Machine’, 1975)

…in the social jungle of human existence there is no feeling of being alive without a sense of identity. (Erikson, 1968: 130)

## Introduction

As stressed already by Aristotle and many influential philosophers after him, humans are deeply social. The importance of social influences for human behaviour was also quite clear to Darwin, as illustrated by his quote above. Modern research shows that social learning and culture have played crucial roles in human evolution since the origin of our species (Henrich, 2015). In fact, social learning and cumulative culture represent some of the most distinctive features of our species (Richerson et al., 2021).

A byproduct of our strong reliance on social learning and conformity with peers was the emergence of novel factors constraining and driving human behaviour: morality, social norms and social institutions. These forces often act against immediate biological or material interests of individuals promoting instead the interests of the society as a whole or of its powerful segments (Singh et al., 2017). Our preferences for social learning, including learning of social norms and beliefs of others, are apparent at very early ages (Whiten, 2017; House et al., 2019). We exhibit striking levels of conformity with opinions of others (Asch, 1951; Jacobs and Campbell, 1961; Bond and Smith, 1996; Cialdini and Goldstein, 2004; Song et al., 2012) and often over-imitate their behaviour even when it is perceivably unrelated to achieving the given goal (Hoehl et al., 2019). We deeply internalise the systems of religious and political beliefs (Gómez et al., 2017) and are strongly influenced by various types of propaganda, e.g. political or commercial (Jowett and O'Donnell, 1992).

Overall, our actions, attitudes and beliefs about others are strongly affected by those of our peers as well as by authorities (educational, cultural, religious, moral, political, administrative, etc). In this paper, by ‘authority’ we mean people who have the power to influence or command thought, opinion, or behaviour of others without necessarily implying the existence of formal institutions behind their power. Peer influence largely works towards reducing variation between interacting individuals and finding consensus around the middle ground (Gavrilets et al., 2016). As a result, conformity with peers usually stabilises behaviour, making changes at the group level difficult. In contrast, an authority's influence can directionally shift actions, attitude and beliefs of a large number of individuals. This is a fact well known to politicians, religious leaders, cultural models, educators, marketing professionals and social media influencers. The resulting effects can be very positive or extremely negative both from individual and group/societal perspectives. For example, certain ‘institutional signals’ can stimulate pro-environment behaviour (Tankard and Paluck, 2016) or can lead to despicable behaviours as studied in Milgram's and Zimbardo's experiments on obedience to authority (Milgram, 1963; Haney et al., 1973). Human history provides plenty of additional examples.

A particularly interesting and important case of an authority's influence is when it acts by persuasion only and uses ‘soft power’ via inculcation and persuasion rather than coercion, punishment and ‘hard power’ and when it promotes (directly or indirectly) actions that can be against the immediate or long-term material interests of individuals. The authority's messaging/propaganda can achieve its goals, for example, directly by modifying individuals’ attitudes towards specific actions and behaviours (Jowett and O'Donnell, 1992; Rozenas and Stukal, 2019; Cantoni et al., 2021) or indirectly by changing individuals’ beliefs about the costs and benefits of their actions (Kuran, 1989; Huang, 2015) or about attitudes and beliefs of their peers (Huang and Cruz, 2021). The authority's messaging is particularly efficient when the authority is viewed as legitimate and its message as trustworthy (Tyler, 1997). The authority can be individual or collective, formal or informal, real or fictitious (e.g. stories of mythical heroes, such as characters in novels or films today, might be influential even if people do not believe they are real).

Here we use mathematical modelling to understand better how the effects of authority on human's attitudes, beliefs and behaviours interact with those of material costs and benefits and with peer influences. The effects of material payoffs on behaviour are usually studied using various flavors of game theory – classical (Fudenberg and Tirole, 1992), evolutionary (Sandholm, 2010), mean field (Tembine, 2017) and quantum (Piotrowski and Sladkowski, 2003). In standard economic models, an individual's preferences are fixed. The effects of peers on individual preferences and opinions are analysed using social influence models focusing on the dynamics of consensus formation (or fragmentation) in social networks as a result of social learning and imitation (Rashevsky, 1949; DeGroot, 1974; Watts, 2002; Friedkin et al., 2016; Zino et al., 2020; Kashima et al., 2021). There are also a few theoretical approaches which consider the joint dynamics of actions/behaviours and opinions/attitudes where the latter are usually understood within the context of personal norms (Kuran, 1989; Rabin, 1994; Kuran and Sandholm, 2008; Gavrilets and Richerson, 2017; Calabuig et al., 2018; Gavrilets, 2020). Some of these models also explicitly account for certain additional psychological phenomena such as cognitive dissonance (Festinger, 1957), social projection (Krueger, 2007) and logic constraints (Friedkin et al., 2016). We will follow this latter tradition, enriching it by explicitly considering the effects of authority and allowing for variation between individuals in various characteristics besides their strategies/actions. Our model is an application of a general theoretical framework recently developed by Gavrilets (2021). In the situations we consider, the authority benefits directly if people respond to its messaging while people cooperate with the authority to avoid psychological costs of non-conformity. In contrast to some approaches where people ‘imitate’ authorities and prestigious individuals (e.g. Henrich et al., 2015), in our models people listen to and adopt the views promoted by the authority.

We will apply our approach to three different phenomena which nevertheless are probably driven by similar underlying processes. The first is cooperative behaviour which has been a major focus of theoretical work in biology and social sciences over the last several decades. The mechanisms and factors that have received most attention in the literature are genetic relatedness, reciprocity, reputation, selective incentives (rewards and punishment) and cultural (group) selection (Nowak, 2006; McElreath and Boyd, 2007; Richerson et al., 2016). We will consider another alternative: people exhibit altruistic tendencies (in real life and in experimental economics labs) mostly because they have internalised such behaviour owing to inculcation by parents, teachers, literature, religion, mass media and other cultural forces in the society. [The ability to internalise norms has evolved because it brings both individual and group-level benefits (Henrich and Ensminger, 2014; Gavrilets and Richerson, 2017).] As a result, people expect themselves and others to behave in a cooperative way and they believe that others have similar expectations. That is, cooperation becomes an injunctive social norm (Bicchieri, 2006; Fehr and Schurtenberger, 2018; Gavrilets and Richerson, 2017; Gavrilets, 2020). As an example, we will use food sharing in small-scale societies which arguably represents one of the most well studied types of cooperation evolved in our species (Marlowe 2009; Gurven and Jaeggi, 2013; Jaeggi and Gurven, 2018). We will show that food sharing can evolve by certain psychological mechanisms responding to elders and prestigious individuals (the authority) as well as other group members pressuring successful hunters to share their kill. In a sense in our model, social learning is ‘hijacked’ to benefit people in a position of authority.

The second phenomenon is the effect of an authority (e.g. a government or a revolutionary leader) on the willingness of citizens to join protests. Social protests have become a constant part of social life throughout the modern world, sometimes leading to revolutions and civil wars (Kuran, 1995). The importance of peer behaviour on individual willingness to engage in protests is well established (Granovetter, 1978; Borna et al., 2016). Also important are the beliefs that individuals have about the attitudes of their peers (Kuran, 1995). For example, even when a majority of people want political changes, political actions can be prevented owing to pluralistic ignorance (Centola et al., 2005), that is, the situation when people mistakenly think that most other people will oppose their personal norms and beliefs. Pluralistic ignorance can lead to preference falsification (Kuran, 1989) when people express preferences that are different from what their true preferences are because they believe they are in minority. People's beliefs and preferences, including political, are also greatly influenced by various types of propaganda (Jowett and O'Donnell, 1992), by biased media (Alesina and Fuchs-Schündeln, 2007; Rozenas and Stukal, 2019) and by education (Bowles and Gintis, 1976; Cantoni et al., 2021). The effects of peers on individual and group behaviour are often described using the Shelling–Granovetter model (Schelling, 1971; Granovetter, 1978) and similar mass-action/threshold approaches (Rashevsky, 1949; Efferson et al., 2020; Gavrilets, 2020), which however assume that individual attitudes remain constant. Here we will extend these approaches by allowing the attitudes to change and by explicitly considering the effects of authority.

The third phenomenon concerns the effects of social identity on behaviour and beliefs of individuals. Social identity is ‘that part of an individual's self-concept that derives from their knowledge of their membership in a social group (or groups) together with the value and emotional significance attached to that membership’ (Tajfel, 1981: 255). Social identity theory postulates that people tend to view themselves as belonging to specific (identity) groups and when they do so, they tend to behave and accept beliefs according to the perceived ‘standards’ of their group. People do so because they receive value from the status of the identity group. Examples of social groups include gender, race, political or professional affiliation; perceived membership in these groups comes with group-specific norms and beliefs. Conformity with those leads to personal pride while deviance causes embarrassment or shame (Tajfel and Turner, 1979; Tajfel, 1981). Priming social identity increasing the importance of the standard of behaviour associated with that social group. When a person's social identity changes, so does that individual's views of the appropriateness of certain actions. To understand better these effects we will adopt our modelling approach by re-interpreting the ‘authority’ as a set of behavioural standards, stereotypes and beliefs associated with a particular group the individual identifies with.

Our main conclusion will be that the effects of authority can provide a simple explanation for a number of important phenomena. Consequently, they need to play much more prominent role in the discussions and empirical and theoretical studies of human social and cultural evolution.

## Models and results

### Modelling framework

Consider a group of individuals. Time is discrete. At each moment, each individual is characterised by an action *x* they take and by an attitude *y* towards possible actions (0 *≤ x, y ≤* 1). If there are just two possible actions, say, *x* = 0 and *x* = 1, then attitude *y* measures their relative subjective appropriateness with high *y* implying high appropriateness of choosing *x* = 1. If there is a continuum of possible actions, then *y* specifies the most appropriate action (i.e. a personal norm, Schwartz, 1977) from the point of view of the individual. An example of the former case would be a decision on whether to participate or not in a mass protest. An example of the latter would a decision on a share of their endowment to donate to a recipient in the Dictator game. Variable *y* is related to the strength of norm internalisation in Gavrilets and Richerson (2017). Our approach can also be viewed as a special case of a more general framework of Gavrilets (2021). Below when talking about attitude *y*, we will occasionally refer to it as ‘belief’ or ‘preference’.

As a result of action *x* the individual gets material utility (i.e. payoff) *π*(*x*), which can depend on the average action 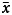 of their group, and psychological utility (i.e. normative value) *V* (*x, y*), which depends on their action *x* and attitude *y* but does not depend on their social environment. We assume the existence of an authority promoting action *x* = *G*. We are agnostic about the source of power of the authority. Rather our model captures its effects by a single parameter *F* measuring efficiency of its messaging which can depend, for example, on the effort or legitimacy of (or trust in) the authority. We postulate that in deciding on the action the individual attempts to maximise utility function *u*:1
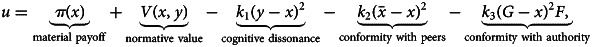
where *k*_1_, *k*_2_ and *k*_3_ are individual-specific non-negative parameters measuring psychological costs owing to sensitivity of decision-making to the corresponding forces. These parameters can depend on specific characteristics of individuals, e.g. cognitive or cultural. For example, individuals reacting negatively to perceived attempts to restrict their freedom (that is, showing psychological reactance) would have low values of *k*_2_ and *k*_3_. Utility function (1) implies that material benefit *π* is traded off against psychological costs owing to cognitive dissonance (i.e. a mismatch between the action *x* and attitude *y*; Festinger, 1957) and mismatch of their action with the average action of their peers, 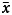, and action *G* promoted by the authority. Note also that eqn (1) implies that individuals condition their actions on the behaviour of their peers (Fischbacher and Gächter, 2010). The conformity term is similar to those emerging in some models of coordination in economics (e.g. Brock and Durlauf, 1999, 2001; Kuran and Sandholm, 2008). In general, *G* can change, e.g. if the authority updates its messaging (Almagro and Andrés-Cerezo, 2010), or the authority could also produce certain goods (e.g. Verdier and Zenou, 2018). However for simplicity we will focus exclusively on messaging and treat *G* as a constant.

We will further postulate that after taking an action, the individual's attitude *y* can change owing to cognitive dissonance and conformity:
2
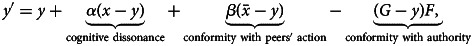

where *α, β* and *γ* are individual-specific non-negative parameters measuring sensitivity of attitudes to the corresponding forces. Here the cognitive dissonance term acts to align individual attitude *y* with a previously taken action *x* (Festinger, 1957; Rabin, 1994; Kuran and Sandholm, 2008; Calabuig et al., 2018). The two conformity terms act to align *y* with the average group behaviour 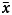 and the action *G* promoted by the authority, respectively. These terms have a simple linear form standard in models of social influence (DeGroot, 1974; Watts, 2002; Centola et al., 2005; Friedkin et al., 2016) and are also related to those used in cognitive neuroscience (Olsson et al., 2020). Note that while individual actions *x* affect material payoffs directly, individual thoughts/beliefs *y* do so only indirectly via subsequent actions.

The change in attitude *y* described by eqn (2) leads to a change in the utility function *u* which in turn can cause subsequent changes in actions taken. We assume that individuals take their actions and update their attitudes synchronously. Between-individual variation in parameters *k*_1_, *k*_2_, *k*_3_, *α*, *β* and *γ* reflects differences in psychology, culture, personal history, information received, etc. Table 1 summarizes model variables and parameters. Note that we do not study the evolutionary origins of this variation (see e.g. Gavrilets and Richerson, 2017) but rather postulate that it is already present and then explore its consequences on relatively short time-scales of individual life spans.

**Table 1.** Main variables, functions, and parameters

	Symbols	Their meaning
Variables	*x y p*	Individual action: *x* = 0 or *x* = 1 Individual attitude (0 ≤ *y* ≤ 1) Frequency of individuals choosing *x* = 1
Functions	*π*(*x*) = *−cx* *V* (*x, y*) = *vxy*	Material payoff Normative value
Parameters	*c*	Material cost of action *x* = 1
	*v*	Measure of normative value of action *x* = 1
	*k*_1_, *k*_2_, *k*_3_	Measures of utility losses specified by eqn (1)
	*α*, *β*, *γ*	Sensitivities of attitudes to different forces specified in eqn (2)
	*G*, *F*	The action promoted by the authority and the efficiency of its messaging

Next we consider three different applications of this model. In the first application, the ‘authority’ will be elders and prestigious individuals in a small-scale society promoting food sharing. In the second case, the authority will be a revolutionary leader promoting mass protests against the government. The third model will consider an imaginary authority – the identity group and its stereotypical behaviour.

For these three cases, we will present analytical approximations capturing the effects of different parameters on the average characteristics of the population at equilibrium. We will use agent-based simulations to illustrate the dynamics of *x* and *y* in time and the resulting distributions of *y* in the population.

### Food sharing in small-scale societies

Consider a group of hunter–gatherers some of whom occasionally succeed in hunting and come into possession of some food items. Other group members express a desire to get a share from successful hunters who can oblige (i.e. choose *x* = 1) and lose food of value *c* or not oblige (i.e. choose *x* = 0). Individuals who share get normative value *vy*, where *v* is a non-negative parameter. Within the group there is an informal authority (e.g. elders who cannot hunt anymore themselves or some other accomplished and prestigious figures) inculcating food sharing among group members (i.e. *G* = 1); the efficiency of inculcation is controlled by parameter *F*. Let *p* be the frequency of sharing. Individuals assume that their peers who do share will disapprove of them if they do not share. Because everybody benefits from sharers, they do not suffer any cost of disapproval. Individuals differ in their attitude *y* towards sharing and in their sensitivity to social influence by peers and authority. We note that in the well-studied Hadza and the Ju’/hoansi there is a lot of bickering and shaming caused by a failure to share (Marlowe, 2009; Wiessner, 2014), which can be interpreted as reflecting individual variation in *y*. This model is described by a special case of eqns (1) and (2) (see the Supplementary Material).

We show in the Supplementary Material, that the deterministic version of this model predicts evolution to a line of equilibria different in *p* and in the distribution of attitudes *y*. At each equilibrium, a proportion of individuals have relatively high values of *y* and share food while remaining individuals have relatively low values of *y* and do not share food. The average difference in attitudes between individuals who share and those who do not is equal to the average of ratio *α/*(*α* + *β* + *γ*), which measures the relative importance of cognitive dissonance in attitude updating process. There can also be equilibria where everybody or nobody shares food. In particular, the state of universal sharing (*p* = 1, *y* = *α* + *β* + *γ*) is stable if the joint effect of normative value, cognitive dissonance, conformity with peers and conformity with authority on decision-making is larger than the benefit lost (*v* + ∑ *k_i_ > c*). The state of no sharing (*p* = 0, *y* = *γ*) is stable if the effects of authority both on actions (*k*_3_) and attitudes (*γ*) are small enough. All model parameters control the location and stability of the line of equilibrium (see the Supplementary Material).

The existence of multiple equilibria in general and of lines of equilibria in particular has two important implications. First, initial conditions become very important, so one needs to study their effects in detail. Second, models with lines of equilibria are structurally unstable, so one needs to study their behaviour under some perturbations.

Below we illustrate the model's behaviour using agent-based simulations which introduce additional features aiming to make the model more realistic. First, we allow for stochasticity in decision-making and in the process of updating the attitudes. The former will be characterised by precision parameter *λ* (large *λ* mean higher precision). The latter is characterised by a standard deviation *σ* of a random perturbation of attitude *y* during the update process (see the Supplementary Material for details). In our numerical simulations, the initial frequency of sharing is set to 0 but we vary the mean 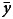 and standard deviation *σ* of the distribution of attitude *y* in the population. Second, we will assume that there is not one but a large number of relatively small groups randomly exchanging migrants at rate *m* (Wright, 1931). This assumption fits many hunting and gathering societies such as the Hudza in north-central Tanzania (Hill et al., 2011). Since our main focus is on the effects of messaging, we choose high enough dispersal rates to reduce the effects of genetic relatedness on the model behaviour.

In agent-based simulations, we set *c* = 1 but allow for most other parameters to differ between individuals. Specifically, for each individual values of *v, k* and *d* are drawn randomly from a ‘broken-stick’ distribution [0, 1]. Similar procedure is used for assigning individual values of parameters *α*, *β* and *γ*. Besides its intuitive appeal, the advantage of the ‘broken-stick’ distribution is that, similarly to the uniform distribution, it does not have any parameters.

**Figure 1.**
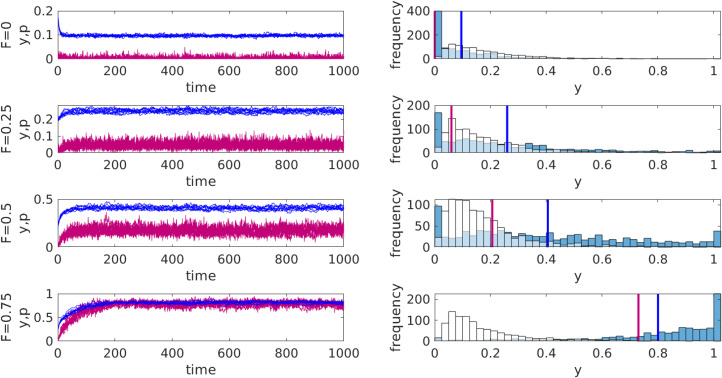
Predictions of the food sharing model for different efficiencies *F* of inculcation promoting sharing (*G* = 1). Left: the dynamics of the average attitude *y* (blue curves) and the frequency of sharers *p* (red curves) for 10 different independent runs for each value of *F*. Right: the initial (white bars) and final (blue bars) distributions of attitudes for one run. The blue and red vertical lines mark the mean attitude 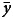 and the frequency *p* of sharers respectively. Log–normal distribution of initial values of *y* with mean 0.2 and standard deviation 0.1. Parameters: benefit lost to sharing *c* = 1, 100 groups of size *n* = 10, precision in decision-making *λ* = 10, standard deviation of error in attitude updating *σ* = 0.05, probability of attitude updating *u_y_* = 0.5, dispersal rate *m* = 0.1, probability of successful hunting *s* = 0.15. Individual parameters *k*_1_, *k*_2_, *k*_3_ and *α, β, γ* are chosen from broken stick distributions on [0, 1] while parameter *v* is set to 1.

Our simulations show that the population quickly evolves to a stochastic equilibrium where a proportion of individuals share food and have high attitudes towards it. The average attitude 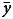 towards sharing is typically smaller than the actual frequency of sharing *p* (because the latter is augmented by the authority's influence). Providing the efficiency of inculcation *F* is high enough, a large proportion of individuals will share food. At the same time, the population as a whole can exhibit a substantial variation in underlying attitudes. Figure 1 illustrates these observations. If authority weakens its messaging, the model predicts that food sharing will be maintained for some time (because it is now internalised) but then it will get weaker and eventually disappear.

### Political protests

Here we will adapt and extend a classical model proposed by Kuran (1989) in his study of unanticipated political revolutions.

Assume that each individual can publicly express the discontent with the current government (e.g. by joining a protest), *x* = 1, or not, *x* = 0. Let *y* be the attitude towards joining the protest (0 *≤ y ≤* 1) and let 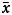 be the perceived frequency of people who are protesting. Kuran (1989) called the variables *x, y* and 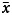 ‘public preference’, ‘private preference’ and ‘collective sentiment’, respectively. He ignored material payoffs (i.e. set *π* = 0) and normative value (i.e. set *V* = 0) but assumed that individuals get an ‘integrity utility’ as well as a ‘reputational utility’. The former depended on the mismatch between the attitude and the action chosen; its effect on utility function is analogous to that of the ‘cognitive dissonance’ term in our eqn (1). The latter was proportional to the frequency of people choosing the same action; its effect is analogous to that of the ‘conformity with peers’ term in our eqn (1).

Let us generalise Kuran's model by postulating the existence of an authority promoting action *G* and assuming that individuals pay certain psychological costs by acting against its message as specified by the last term in eqn (1). If the focal authority is the government, then *G* = 0. If the focal authority is the opposition, then *G* = 1. Parameter *F* measuring the efficiency of the authority's messaging (propaganda) depends on the perceived trust/legitimacy of the authority and on its effort. Kuran (1989) assumed that individual attitudes *y* were constant in time. In contrast, we postulate that after taking the actions and observing peer behaviour, individuals update their attitudes according to eqn (2).

**Figure 2.**
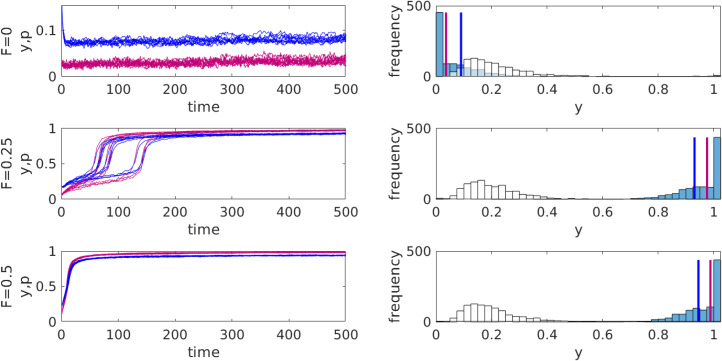
Predictions of the political protests model for different intensities of propaganda *F* promoting protests (*G* = 1). Left: the dynamics of the average attitude *y* (blue curves) and the frequency of protesters *p* (red curves) for 10 different independent runs for each value of *F*. Right: the initial (white bars) and final (blue bars) distributions of attitudes for one run. The blue and red vertical lines mark the mean attitude 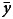 and the frequency *p* of protests respectively. Log–normal distribution of initial values of *y* with mean 0.2 and standard deviation 0.1. Parameters: population size *n* = 1000, precision in decision-making *λ* = 10, standard deviation of error in attitude updating *σ* = 0.05, probability of attitude updating *u_y_* = 0.5.

This model is similar to the one describing food sharing (except that here *b* = 0 and that we consider a well-mixed population with individuals knowing the frequency of protesters 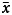). We illustrate its behaviour using agent-based simulations which allow for stochasticity in decision-making and in updating attitudes. Figure 2 shows that the propaganda by a revolutionary leader can change the distribution of attitudes in the population and cause, if sufficiently strong, widespread protests. In contrast if we assume that attitudes do not change, then for the initial distribution of *y* and parameter values used in Figure 2, the frequency of people joining the protests will remain very low for all values of propaganda efficiency *F* considered in spite of a significant hidden discontent. Figures S1 in the Supplementary Material shows that the system can have multiple equilibria so that the eventual outcome of the dynamics can depend on the initial distribution of attitudes.

We can also consider the case when there are multiple alternative authorities in the society. For example, let one authority promote protests (i.e. *G* = 1) with efficiency *F*_1_ and another promote acquiescence (i.e. *G* = 0) with efficiency *F*_0_. This model is mathematically equivalent to that with a single authority promoting the value of *G* = *F*_1_*/*(*F*_0_ + *F*_1_) with efficiency *F* = *F*_0_ + *F*_1_. Figure S2 in the Supplementary Material shows that if the propaganda efficiency of the government matches that of the opposition (i.e. *F*_0_ = *F*_1_ so that *F* = 0.5), the protests are suppressed. This may explain why even regimes capable of severe repression nonetheless devote substantial resources to persuasion.

### Social identity and altruism

Numerous studies have shown that priming social identity affects human decisions and preferences. For example, making ethnic or racial identity more salient affect time and risk preferences (Benjamin et al., 2010). Making religious identity more salient decreases contributions to public goods of Catholics but increases it for Protestants, and increases agnostic/atheist risk aversion (Benjamin et al., 2016). Chang et al. (2019) used a series of Dictator games described with either politically charged tax or neutrally framed language. They observed that subjects’ political identities interact with these frames, causing changes in both norms and choices. In particular, framing made Democrats prefer equalised outcomes, and Republicans reluctant to redistribute payments even when it leaves them disadvantaged. Using surveys conducted in UK and US, Pickup et al. (2021) showed that individuals were aware of the norms attached to their political identities, were knowingly choosing norm-compliance at personal cost, and that their costly expression of political identity varied with norm salience and strength.

Within our framework, the effects of social identity can be formalised as emerging from messaging of an imaginary authority – the identity group. Parameter *F* then can be interpreted as measuring the salience of social identity while *G* as a stereotypical behaviour associated with the focal social identity group. To be specific, consider the Dictator game which is often used to study cooperative and altruistic behaviour with identity considerations (e.g. Chang et al., 2019). Let a focal individual make a decision on a share *x* of their endowment *b* to transfer to a partner. Then the material payoff term in eqn (1) is *π* = *b*(1 *−x*). Note that in this model all interactions are dyadic rather than are happening in a group setting. As above, let *y* be the individual's attitude, i.e. the most appropriate, from their personal perspective, proportion of the benefit to be shared under given circumstances. Let us ignore the ‘conformity with peers’ terms in eqns (1) and (2) because subjects often were not informed about peer behaviour. This complete the formulation of the model.

We show in the Supplementary Material that, given *y*, the best response action/donation is a weighted sum of the personal norm *y* and the action *G* promoted by the authority:3
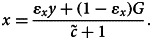
where parameter 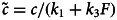 is the strength of material payoffs relative to that of cognitive factors in the utility function and parameter *ɛ_x_* = *k*_1_*/*(*k*_1_ + *k*_3_*F)* measures the relative strength of the effects of cognitive dissonance on actions. Computing the derivative of *x* with respect to *F*, one can see that donation *x* to the partner increases with identity salience *F* if
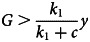


(that is, if stereotypical donation *G* is large enough relative to individual attitude *y*) but decreases with *F* otherwise. Thus, as argued by Benjamin et al. (2010), this model predicts that priming people experiencing strong cognitive dissonance (i.e. with large *v*) can cause negative effect on *x*.

However one can also expect that personal norms *y* can change as a result of psychological processes. Without priming (i.e. if *F* = 0), eqn (2) shows that *y* will evolve to match *x* after which *x* will evolve to zero so that individuals will not transfer any funds to the partner. In contrast, with priming *F*, at equilibrium,4*a*
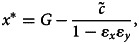
4*b*
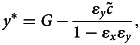
where parameter *ɛ_y_* = *α/*(*α* + *γF*) measures the relative strength of the effects of cognitive dissonance beliefs (see the Supplementary Material). Equations (4) show that *x^∗^* and *y^∗^* are both smaller than the group's standard *G* with the personal norm *y* being closer to *G* than *x^∗^* (so that *G − y^∗^* = *ɛ_y_*(*G − x^∗^*)). Increasing the salience *F* of social identity decreases 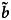, *ɛ_x_* and *ɛ_y_* moving *x^∗^* and *y^∗^* close to the identity standard *G*. Increasing the cost of expressing identity 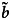 or the strengths of cognitive dissonance in decision-making, *ɛ_x_*, and belief formation, *ɛ_y_*, have opposite effects.

The derivatives of *x^∗^* and *y^∗^* with respect to *F* are always positive meaning that priming always has positive effects on actions and beliefs. This conclusion is different from that above for the model where beliefs were constant (also see Benjamin et al., 2010). On a short time-scale, some people with a very high personal norm *y* and experiencing significant cognitive dissonance (large *c*) may donate money to the partner even in the absence of priming (i.e. if *F* = 0). However on larger time scales, our model predicts that without a continuous priming *y* will evolve to match *x* and then contributions will disappear.

## Discussion

Human decision-making in social interactions is clearly controlled not only by material cost–benefit considerations, but also by other factors including their values and beliefs, social influences and errors. Importantly, humans differ in various psychological characteristics and in their beliefs and values. The latter can dynamically change coevolving with their actions (Tormos, 2020; Szekely et al., 2021; Levy, 2021; Tverskoi et al., 2022). Among various social influences those by external authorities (e.g. cultural, religious, political, moral, administrative, individual or group, formal or informal, real or fictitious, etc.) are particularly important. The messaging of external authorities affects not only individual actions but also their beliefs. While the effects of peer influences have been extensively studied and modelled in the field of cultural and social evolution, those of external authorities have not received much attention. Here we have argued here that these effects can explain certain puzzling features of humans, such as large-scale cooperation with unrelated individuals, the emergence and evolution of social norms and some other types of mass behaviour. Our argument is supported by a unifying modelling framework accounting for various factors of human decision-making and psychological differences between individuals. Most of earlier related modelling work focuses exclusively either on actions taken by individuals (using game-theoretic approaches) or on publicly expressed opinions (using models of social influence). In contrast, we used a more general approach in which actions coevolve with the degree to which individuals internalise/value particular actions (Gavrilets and Richerson, 2017; Calabuig et al., 2018; Gavrilets, 2021). Our approach accounts for some psychological processes and forces affecting actions, attitudes and beliefs, such as cognitive dissonance (Festinger, 1957), conformity with peers (Song et al., 2012), social identity (Tajfel and Turner, 1979; Tajfel, 1981) and obedience to authority (Milgram, 1963; Haney et al., 1973). We have illustrated our approach using three different phenomena.

### Food sharing

The norm of food-sharing is common in small-scale societies and represents probably one of the most ancestral type of altruistic/cooperative behaviour in humans (Marlowe, 2009; Gurven and Jaeggi, 2013). Explanations of food-sharing include nepotism (i.e. sharing with kin), reciprocity (i.e. sharing is conditional on past receipt of food), costly signalling (to display the donor's desirable qualities), and tolerated scrounging (when giving up some food helps avoid potential physical or reputational costs from hoarding) (Kameda et al., 2003; Marlowe, 2009; Gurven and Jaeggi, 2013). These mechanisms have been studied using standard game-theory models comparing the long-term payoffs of different strategies (Jaeggi and Gurven, 2018). The overall conclusion of earlier work is that all the above factors may be in play with tolerated scrounging being probably most consistent with data (Kameda et al., 2003; Marlowe, 2009; Jaeggi and Gurven, 2018).

The logic of our approach is closely related to that of tolerated scrounging. However rather than demonstrating that food-sharing strategy can lead to higher material payoffs, we show that individuals can develop intrinsic preference for sharing to avoid psychological costs of disapproval by elders and peers. The pressure from the later to share is both ‘cheap’ and is directly motivated by a hope to receive a share of the kill. The latter means that sharing demands are not necessarily a ‘public good’, so that there is no second-order free-rider problem (Henrich and Boyd, 2001; Boyd et al., 2003) which can prevent the evolution of cooperation. That is, the norm of food-sharing can evolve without relatedness, reciprocity, reputation, or group selection once the proximate mechanisms of social influence have evolved to be sufficiently important. Here we do not model the ultimate evolutionary mechanisms that give rise to an important role for social influence, such as Darwin's idea that prosocial emotions evolved by tribal-scale selection for cooperation (Richerson et al., 2016). Henrich and Gil-White (2001) argued that social influences including those of prestigious individuals are a result of psychological adaptations that evolved to improve the quality of culturally acquired information.

In small-scale societies moral inculcation takes place systematically and repeatedly starting with early age. These happen during day-to-day activities (Wiessner, 2005), during certain ritual such as male initiation or the trance dance (Wiessner and Tumu, 1998), and during evening-time conversations in stories told by elders and leaders (Wiessner, 2005; Smith et al., 2017). In small-scale societies, there are usually multiple types of authorities corresponding to different domains of everyday life, e.g. fishing, growing yams and using medicine (Henrich and Broesch, 2011). Parents and older relatives play an important role in promoting helping (Clark, 2021). Children's responsiveness to social norms (House et al., 2019; Hoehl et al., 2019) and the beliefs (Kovács et al., 2010) of others develops similarly across small-scale horticulturalists to large urban societies (House et al., 2019).

Cross-cultural experimental economics studies show significant differences between small-scale societies in their cooperativeness (Bowles et al., 2001). This variation is viewed as reflecting differences in optimal behaviour between environments (Lamba and Mace, 2011). However it is also possible that differences in cooperativeness reflect differences in the degrees of acceptance of an authority across societies.

Many social norms are group beneficial. Our approach applies to norms from which the authority benefits equally or more than regular group members. The cases where social norms are detrimental to the authority (e.g. individual preferences for egalitarianism in nonegalitarian societies) fall outside of our framework.

### Political movements and revolutions

Many mathematical models of of political protests, riots, or revolutions are related to the classical Shelling–Granovetter model (Schelling, 1971; Granovetter, 1978). In that model, each individual is characterised by their own conformity ‘threshold’ defined as a minimum frequency of a particular behaviour (e.g. join the riot) in the population needed for the focal individual to adopt the same behaviour. The ‘thresholds’ are constant in time, and the transition to mass protests is triggered by individuals with low thresholds with others subsequently joining in. Instead of rather abstract ‘thresholds’, later work started to use the formalism of utility functions explicitly accounting for various factors affecting decision-making (Kuran, 1989; Gavrilets, 2020). This is the approach we have taken here, generalising it by allowing for individual values to change (which leads to changes in the individual ‘thresholds’) and by explicitly accounting for the effects of external authorities.

Our results show that accounting for changes in individual values/beliefs leads to qualitative and quantitative changes in model predictions. In particular, the model predicts an increased stability of *status quo* owing to increasing alignment of beliefs with actions relative to what can be expected if beliefs do not change. In a sense, changes in beliefs lead to increasing individual ‘thresholds’, so that the revolutionary change becomes more difficult. A crucial component of Kuran's (1989, 1995) theory is the notion of ‘preference falsification’ – that is, a mismatch between personal beliefs and actual behaviour. Our model suggests a reduced proportion of individuals falsifying their preferences than what can be expected with stable attitudes. This means the society is more resistant to change. Our results further highlight the importance of messaging/propaganda by trusted/legitimate authority in changing the status quo.

### Priming social identity

Identifying with a particular social group often comes with a set of behavioural standards (Tajfel, 1981; Suhay, 2015; Smaldino, 2019). Adherence to in-group norms is critical for a status among in-group peers (Suhay, 2015). Individuals are aware of the norms attached to their social identities (e.g. political, Pickup et al., 2021). They will comply with the norms at personal cost; the extent of their compliance varies with norm salience and strength (Pickup et al., 2020, 2021). Partisan identity affects not only individual actions but also their beliefs, e.g. on climate change (Doell et al., 2021). Individuals also exhibit significant and robust differences in ‘groupiness’ across different social situations. For some individuals, just being in a group setting strongly influences their thoughts and actions (Kranton et al., 2021). One of our goal here was to capture these effects in a mathematical model and study their consequences.

Applying our approach to the settings of the Dictator game, which is often used to study the effects of social identity on cooperation, we have shown that, without priming, individuals are expected to stop transferring funds to the partner. With priming, they will value transferring some funds and will do so. Individual actions and beliefs will be controlled by three composite parameters, one of which measures the amount of material benefit relative to the strength of psychological factors (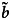) and two of which measure the relative effects of cognitive resonance on actions and beliefs (*ɛ_x_, ɛ_y_*). Individuals’ beliefs are always closer to the group standard *G* than their average action. The extent of mismatch between actions *x* and beliefs *y* is controlled by the cognitive dissonance parameter *ɛ_y_*. Earlier Benjamin et al. (2016) developed and then tested a model predicting the effects of social identity on decisions in a single round of social dilemmas. Our results show that their conclusions will probably not hold over multiple rounds.

Here we took the existence of authorities as given. The current thinking is that human groups have ‘authorities’ because of the differences in various types of knowledge and power between individuals/groups which come from the differences in their traits/characteristics. We note that leadership is also common in non-human animals where ‘leaders’ emerge for similar reasons (Smith et al., 2016). We have also interpreted a set of behavioural standards, stereotypes and beliefs associated with a particular group the individual identifies with as an impersonal or fictitious authority. Hogg (2001) and others (e.g. Haslam et al., 2011) have argued that these stereotypes often influence who will most likely emerge as real authority and leaders – specifically, most prototypical group members. More prototypical leaders are also perceived to be more effective leaders. That authorities can spread certain norms is one of their emergent features.

For each of the three specific applications considered, we showed that our approach leads to different (or simpler) explanations of human behaviour than alternative models. Which mechanism better describes what is happening in reality is an empirical question. In all these models we identify and explicitly characterise the degree of mismatch between actions and attitudes which could not be done in standard models ignoring the dynamic nature of attitudes.

We have treated the efficiency of messaging in a parsimonious way by using a single parameter *F*. In reality, *F* will depend on details of the system under study. It can be weaker or stronger depending on the group size, the type of authority (distributed or concentrated), the effort put by the authority, its legitimacy, the consistency of messaging, etc. For example, in ‘Big Man’ systems, authority is partially based on the charisma of individuals who can influence followers from a kin group outwards of perhaps a few hundred individuals (Wiessner and Tumu, 1998). In conical clans, the chiefs can command thousands of individuals (Kirch, 1984). In modern societies the reach of, say, cultural authorities active online can potentially extend to tens of millions. We note the question of how effective different types of authorities using ‘soft’ power are is largely an open empirical question.

One practical application of our approach towards quantifying the effects of authority is to estimate parameters *v*, *k*_1_, *k*_2_, *k*_3_, *α*, *β* and *γ* of our model. This can be done by fitting linear regressions describing best response actions (eqn S1 in the Supplementary Material) and attitude dynamics (eqn (2)). The data could be obtained by measuring individual actions simultaneously with eliciting their attitudes and beliefs (d'Adda et al., 2020; Górges and Nosenzo, 2020; Andreozzi et al., 2020; Szekely et al., 2021; Tverskoi et al., 2022). An interesting question is whether the estimated parameters *k*_3_ and *γ* (representing the impact of conformity with authority) are large or small compared with the coefficients for other terms in an observed population. We expect that messaging will mostly work indirectly through changing attitudes *y* (as controlled by parameter *γ*) rather than by changing actions directly (as controlled by parameter *k*_3_). Also it would be important to know whether the coefficients vary by population (e.g. whether they depend on its culture) or by the kind of action. Our model postulates that different terms combine additively. If this proves wrong, structural modifications to the model might be needed (e.g. with the terms combining multiplicatively).

Obviously, our approach could be too simple, and the way it breaks down would lead to more complex but more realistic models. In the models studied here, increasing messaging or priming increases the frequency of the focal behaviour. However in related models with more complex (nonlinear) payoff functions, messaging can ‘backfire’, i.e. result in the opposite effect (Gavrilets, 2021). Although our analysis focused on rather specific models, our results should be applicable more generally. For example, the payoffs in the food sharing model correspond more generally (up to rescaling) to the payoffs of, e.g. cooperators and defectors in a linear public goods game, where selfish actions dominate in the absence of additional factors. At the same time, our approach has certain limitations which need to be removed in future work. In particular, in our models, we assumed that different factors interact in an additive way. Although this assumption is a simple and natural first step, nonlinear interactions are also possible. For example, Bowles (2016) describes a ‘crowding-out’ effect in a day-care centre in Israel where an introduction of fees for being late to pickup a child resulted in an increased frequency of parents violating the norm. Also, our equations for the change in personal attitude do not depend on material factors. However, individual attitudes can depend on the payoffs received, e.g. via the phenomenon of effort justification (Festinger, 1957; Pickup et al., 2020). In our models, individuals react to the current frequency of a particular behaviour among peers. However some data show that people also react to the rate of change in the frequency of behaviour (Sparkman and Walton, 2019). We assume that the authority's messaging is constant, while the authority can change it depending on the situation (Almagro and Andrés-Cerezo 2010). On the other hand, the linearity of our model implies that its parameters can be readily estimated using e.g. behavioural economics experiments, such as those in Szekely et al. (2021) and *Tverskoi et al. (2022)*.

Overall, our model offers insights into the complex interactions of actions and beliefs promoted by cultural authorities strengthening our intuition but also providing additional insights on the effects of parameters and changing attitudes. It demonstrates that capturing some neglected aspects of human psychology and social dynamics in the model can lead to different (or simpler) explanations of certain aspects of human behaviour. It also can serve as a guide to the kinds of information that an empirical study would need to consider to isolate the effect of authority's influence on a decision alongside other basic influences. Our results suggest that the effects of external authorities, of changing beliefs, and of differences between people must be included in mathematical models, studied empirically and accounted for when developing different policies aiming to modify or sustain human behaviour.

## Data Availability

The Matlab code used is available from https://volweb2.utk.edu/~gavrila/open/sharing-open.m and https://volweb2.utk.edu/~gavrila/open/kuran-open.m

## Supplementary material

For supplementary material accompanying this paper visit https://doi.org/10.1017/ehs.2022.48. click here to view supplementary material
