# Supplementary material for: Authority matters: propaganda and the coevolution of behaviour and attitudes
Source: Evol Hum Sci. 2022 Oct 28;4:e51. doi: 10.1017/ehs.2022.48 (PMC10426013; doi:10.1017/ehs.2022.48)
Supplement: Supplementary file 1 [file S2513843X22000482sup001.pdf]

Supplementary Information

Norm internalization and behavior: inculcation, propaganda, and priming social identity

Sergey Gavrilets and Peter J. Richerson

Food sharing model

Dynamics predicted by equations (1-2) of the main text. Using equation (1) with $\pi = -cx$ and $V = vyx$, we can find the corresponding utilities of two actions, $u(1)$ and $u(0)$. With $GF = 1$, their difference $\Delta u = u(1) - u(0)$ for a focal individual can be written as

$$\Delta u = -c + vy + k_1(2y - 1) + k_2(2p - 1) + k_3. \quad \text{(S1)}$$

Individuals for whom $\Delta u > 0$ will share food (i.e., choose $x = 1$) while those for whom $\Delta < 0$ will not.

Let there be an equilibrium with a frequency p of food-sharers. Consider equation (2). We will abuse notation by assuming that the values of α, β and γ are normalized so that their sum is one for each individual. Then the attitudes of individuals with $x = 0$ and $x = 1$ are

$$y_0 = \beta p + \gamma,\ y_1 = \beta p + \gamma + \alpha.$$

Taking the average of y in these two subgroups of individuals, we see that

$$\bar{y}_1 - \bar{y}_0 = \bar{\alpha}.$$

That is, the average difference in attitudes between individuals who share and those who do not is equal to the average relative importance $\overline{\alpha}$ of the cognitive dissonance.

The state of universal sharing (i.e., $p = 1$ and $y = 1$) is stable if at this state $\Delta u > 0$ for all individuals. From equation (S1), this is equivalent to condition

$$v + k_1 + k_2 + k_3 > c,$$

for all individuals. That is, the joint effect of normative value, cognitive dissonance, conformity with peers and conformity with authority on decision-making is larger than the benefit lost.

The state of no sharing (i.e., $p = 0$ and $y = \gamma$) is stable if $\Delta u < 0$ for all individuals. From equation (S1), this is equivalent to condition

$$c + k_1 + k_2 > \gamma(v + 2k_1) + k_3,$$

for all individuals. That is, the joint effect of conformity with authority on decision-making and beliefs is smaller than the benefit lost plus the effects of conformity with peers and cognitive dissonance.

From equation (S1), the equilibrium with an intermediate value of p is stable with respect to decision-making if for all individuals with $x = 0$, $\Delta u < 0$, while for all individuals with $x = 1$, $\Delta u > 0$. Solving for p, we find that stability requires that

$$\frac{c + k_1(1 - 2\gamma - 2\alpha) + k_2 - k_3 - v(\alpha + \gamma)}{\beta + 2k_1\beta + 2k_2} \equiv p_{\min} < p < p_{\max} \equiv p_{\min} + \frac{\alpha}{\beta + \frac{2k_2}{2k_1 + v}}$$

for each individual. This condition can be written as

$$\max(p_{\min}) < p < \min(p_{\max}),$$

where maximum and minimum are computed over the whole population. If the above condition if satisfied, it defines a line segment each point p of which is an equilibrium. Each equilibrium is characterized by variation in individual attitudes y.

Agent-based simulations. As mentioned in the main text, to study this model numerically we introduce some additional features in an attempt to make it more realistic. First, we assume that there is not one but a large number of relatively small groups of size n between each individuals randomly move at rate m (using the classical island model, Wright (1931)).

Second, we allow for stochasticity in decision making and in the process of updating the attitudes. In making decisions, individuals aims to maximize the utility. To capture the errors in utility evaluation, we assume that individuals choose $x = 1$ or $x = 0$ with probabilities S and $1 - S$, respectively, where S is increasing with Δu. Specifically, following the Quantal Response Equilibrium (QRE) approach (Goeree et al. 2016), we set

$$S = 1/(1 + \exp(-\lambda \Delta u)), \tag{S2}$$

where λ is a non-negative precision parameter. For example, if $\lambda = 0$ (zero precision), then $S = 0.5$ and individual make random decisions; if $\lambda = \infty$ (infinite precision), individuals always chooses the action maximizing utility ($x = 1$ if $\Delta u > 0$ and $x = 0$ if $\Delta u < 0$). The advantage of the QRE approach over alternatives (e.g., Young 1998) is that the magnitude of errors decreases as Δu becomes larger.

We introduce stochasticity in the attitude updating by adding a random perturbation (with zero mean and a small standard deviation ε) to the right hand side of equation (2).

Third, we assume that a random proportion s of individuals are successful hunters. Their attitudes are updated according to equation (2) of the main text. For remaining individuals, the changes in attitudes y are described by the same equation (2) except that the cognitive dissonance term is removed (because for these individuals there is no action taken).

The order of events in numerical simulations is 1) decision making, 2) attitudes updating, 3) random dispersal of a proportion m of individuals.

Political protests model

Mathematically, this model is similar to that of food sharing except that, following Kuran (1989) we ignore material payoffs (i.e. set $c = 0$) and normative value (i.e. set $V = 0$). In particular, the deterministic version of the model can have a line of equilibrium with different values of p. At each equilibrium, there will be some distribution of attitudes in the population.

The order of events in numerical simulations is 1) decision making and 2) attitudes updating.

Social identity model

Because in this model individual action x is a continuous variable, finding the best response requires a different procedure. Specifically, differentiating utility function (1) with respect to x we find that given y, the value of x maximizing u is

$$x = \frac{k_1 y + k_3 FG - c}{k_1 + k_3 F} = \varepsilon_x y + (1 - \varepsilon_x) G - \tilde{c},$$

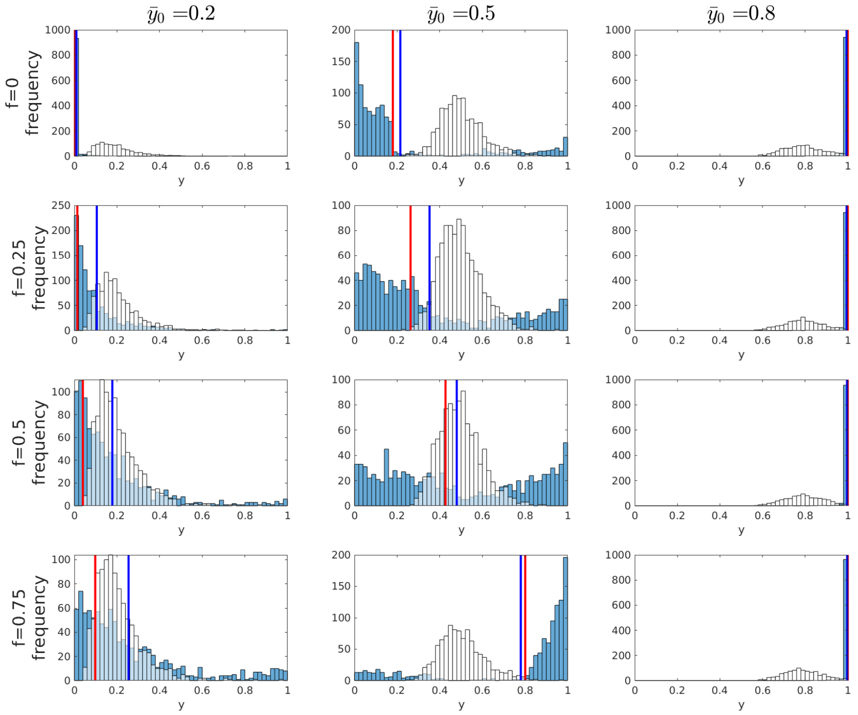

Figure S1: Effects of initial distribution of y in the political protests model for different intensities F of an authority message promoting protests ($G = 1$). The initial (white bars) and final (blue bars) distributions of attitudes for one run. The blue and red vertical lines mark the mean attitude $\bar{y}$ and the frequency p of protests, respectively. Parameters: $k_{p,\max} = k_{g,\max} = 0.75, V = 0.1$. Lognormal distribution of initial values of y with mean 0.2 and standard deviation 0.1. A population of size $n = 1000$. Deterministic updating with no stochasticity ($\lambda = \infty, \sigma = 0.0, u_y = 1$).

where $\varepsilon_x = k_1/(k_1 + k_3 F)$ and $\tilde{c} = c/(k_1 + k_3 F)$. From equation (3), we find that at equilibrium,

$$y = \frac{\alpha x + \gamma F G}{\alpha + \gamma F} = \varepsilon_y x + (1 - \varepsilon_y) G,$$

where $\varepsilon_y = \alpha/(\alpha + \gamma F)$. Solving the two above equations, we find the expressions (4) for the equilibrium values of x and y.

The order of events in numerical simulations is 1) decision making and 2) attitudes updating.

References

Goeree, J., Holt, C., and Palfrey, T. (2016). Quantal Response Equilibrium: A Stochastic Theory of Games. Princeton University Press, Princeton, NJ.

Young, E. P. (1998). Individual Strategy and Social Structure: An Evolutionary Theory of Institutions. Princeton University Press, Princeton, N.J.

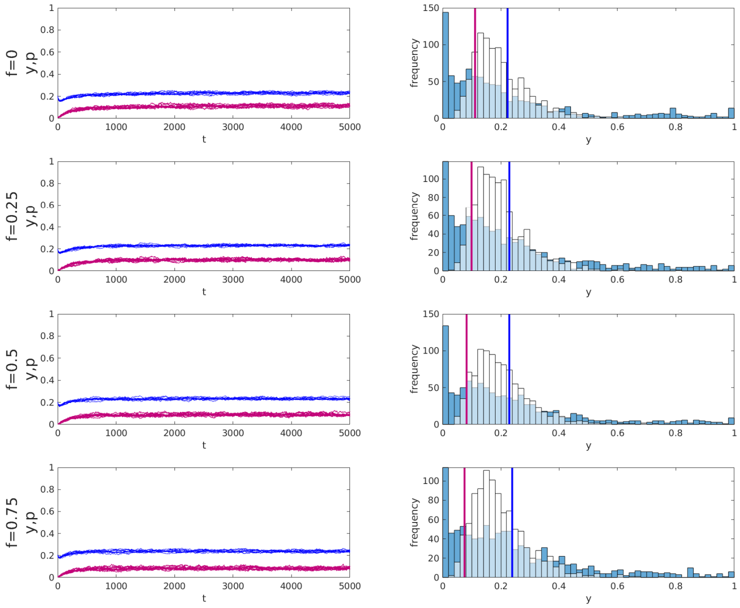

Figure S2: Predictions of the political protests model for different equal intensities F of the government and opposition messaging (so that $G = 0.5$) Left: The dynamics of the average attitude y (green curves) and the frequency of protesters p (red curves) for 10 different independent runs for each value of F. Right: the initial (white bars) and final (blue bars) distributions of attitudes for one run. The blue and red vertical lines mark the mean attitude $\bar{y}$ and the frequency p of protests respectively. Parameters: $k_{p,\max} = k_{g,\max} = 0.75, V = 0.1$. Lognormal distribution of initial values of y with mean 0.2 and standard deviation 0.1. A population of size $n = 1000$, precision $\lambda = 100$, error $\sigma = 0.05$, probability of attitude updating $u_y = 0.5$.
